# Islet autoimmunity and progression to type 1 diabetes in the Finnish DIPP study: comparison between genetically susceptible children with and without an affected first-degree relative

**DOI:** 10.1007/s00125-025-06573-6

**Published:** 2025-11-08

**Authors:** Salla Kuusela, Jaakko J. Koskenniemi, Toni Valtanen, Tytti Pokka, Taina Härkönen, Jorma Ilonen, Johanna Lempainen, Anni Kyrönniemi, Jorma Toppari, Mikael Knip, Päivi Keskinen, Riitta Veijola

**Affiliations:** 1 Department of Pediatrics, Research Unit of Clinical Medicine, Medical Research Center, University of Oulu, Oulu, Finland; 2 Department for Children and Adolescents, Tampere University Hospital, Tampere, Finland; 3 Population Health Research Centre, University of Turku and Turku University Hospital, Turku, Finland; 4 Department of Pediatrics, Turku University Hospital, Turku, Finland; 5 Department for Children and Adolescents, Medical Research Center, Oulu University Hospital, Oulu, Finland; 6 Research Service Unit, Oulu University Hospital, Oulu, Finland; 7 Research Program for Clinical and Molecular Metabolism, Faculty of Medicine, University of Helsinki, Helsinki, Finland; 8 Institute of Biomedicine, Immunogenetics Laboratory, University of Turku, Turku, Finland; 9 Institute of Biomedicine, Research Centre for Integrative Physiology and Pharmacology, University of Turku, Turku, Finland; 10 InFLAMES Flagship Research Centre, University of Turku, Turku, Finland; 11 Pediatric Research Center, New Children’s Hospital, Helsinki University Hospital, Helsinki, Finland; 12 Tampere Center for Child Health Research, Tampere University Hospital, Tampere, Finland

**Keywords:** Children, Family history, First-degree relative, Genetic risk, Islet autoimmunity, Type 1 diabetes

## Abstract

**Aims/hypothesis:**

Islet autoimmunity during presymptomatic type 1 diabetes is heterogeneous. We hypothesised that a positive family history of type 1 diabetes is associated with specific characteristics of the autoimmune process resulting in clinical diabetes. In a prospective birth cohort study, we compared the initiation and evolution of islet autoimmunity and the rate of progression from islet autoimmunity to diabetes between children with and without a first-degree relative (FDR) with type 1 diabetes.

**Methods:**

In the Finnish Type 1 Diabetes Prediction and Prevention (DIPP) study, we prospectively followed children with HLA-conferred susceptibility from birth for the appearance of islet autoantibodies (IAA, GADA, IA-2A, ZnT8A), further development of islet autoimmunity, and progression to clinical diabetes. The presence of type 1 diabetes among their FDRs was recorded at the time of birth, and the family history data was updated during the follow-up period.

**Results:**

Among a total of 1334 children with confirmed positivity for at least one islet autoantibody, 145 (10.9%) had one or more FDRs with type 1 diabetes at the time of birth (FDR+). During a median follow-up period of 8.6 years, FDRs of an additional 87 children developed type 1 diabetes (FDR− FDR+). At seroconversion, both FDR+ and FDR− FDR+ children were significantly more often positive for GADA and multiple autoantibodies than children without affected FDRs (FDR−). The seroconversion age was similar between the three groups (median 2.7 vs 2.1 vs 3.0 years in FDR+, FDR− FDR+ and FDR− children, respectively). During the follow-up period, FDR+ and FDR− FDR+ children more often had IAA, GADA, IA-2A and multiple autoantibodies than FDR− children, and progressed more frequently to diabetes (55.9 vs 57.5 vs 38.9%, respectively). Time from seroconversion to clinical diabetes was significantly shorter in FDR+ children compared with FDR− children (2.7 vs 3.6 years). Children with paternal type 1 diabetes at birth (*n*=71; i.e., the father had type 1 diabetes) were twice as often positive for multiple autoantibodies at seroconversion as those with maternal type 1 diabetes (*n*=50; i.e. the mother had type 1 diabetes) (39.4% vs 20.0%).

**Conclusions/interpretation:**

At seroconversion, genetically susceptible children who had one or more FDRs with type 1 diabetes, especially an affected father, were more often positive for GADA and multiple islet autoantibodies. During the follow-up period, children with an affected FDR were more often positive for IAA, GADA and IA-2A, and progressed to clinical type 1 diabetes more often and faster than children without an affected FDR. These data should be considered when designing intervention and screening studies.

**Graphical Abstract:**

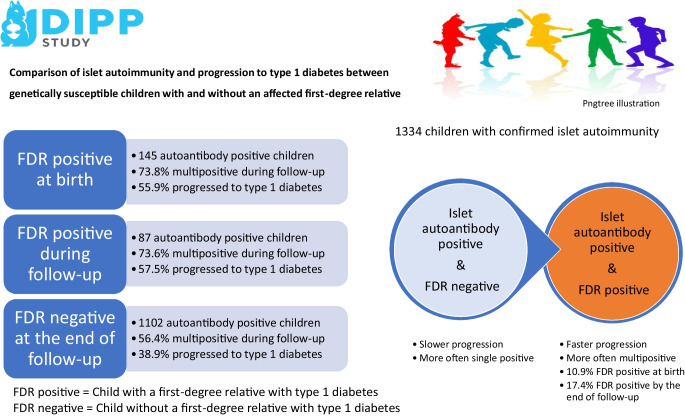

**Supplementary Information:**

The online version contains peer-reviewed but unedited supplementary material available at 10.1007/s00125-025-06573-6.


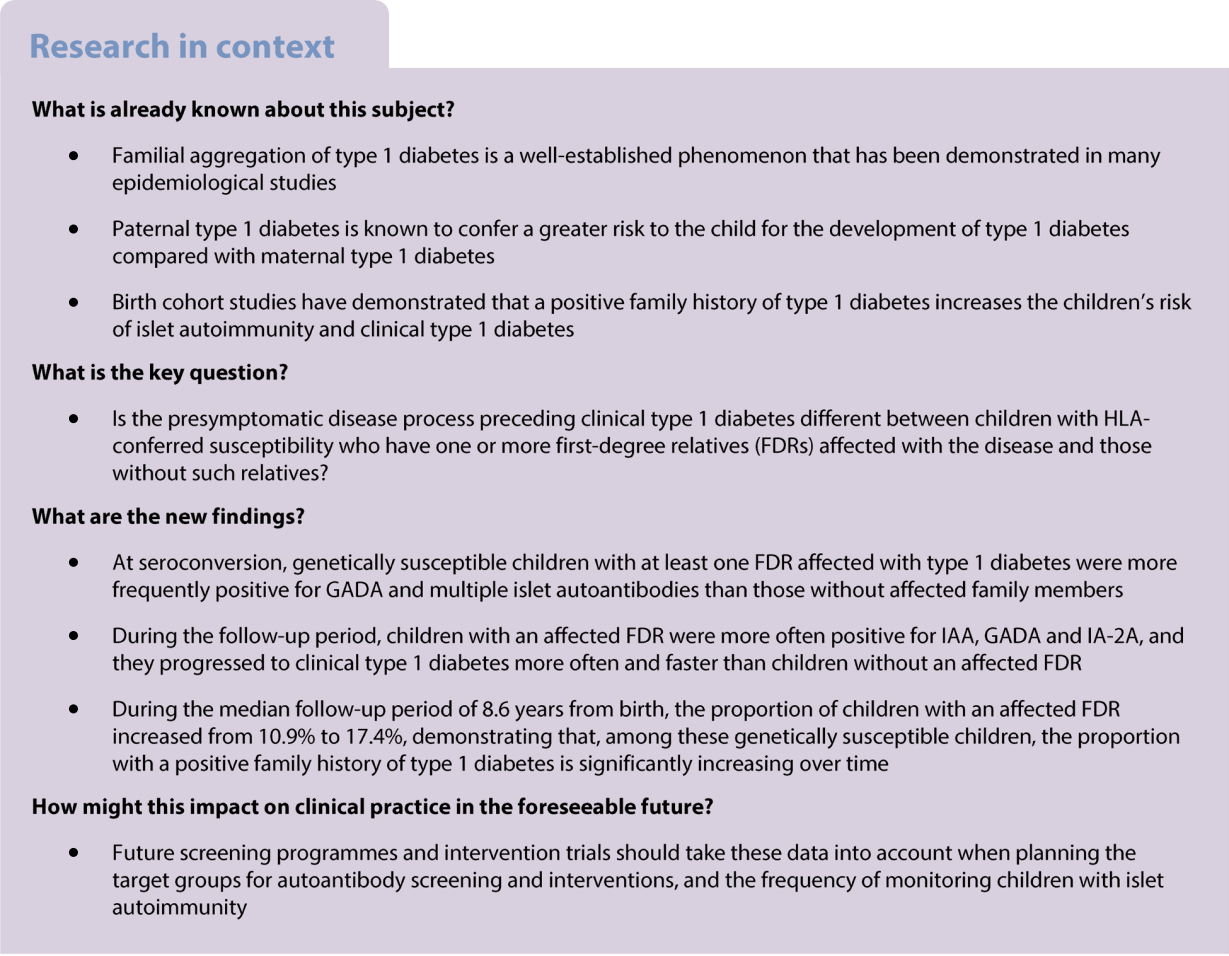


## Introduction

Familial aggregation of type 1 diabetes is a well-established phenomenon that has been demonstrated in many epidemiological studies over the decades [1–4]. The offspring of patients with type 1 diabetes have a 10-fold excess risk of developing the disease compared with the general population in Finland [5]. If the child has a father or a sibling with type 1 diabetes, his/her risk of developing the disease is two times higher than that of a child whose mother has type 1 diabetes, but the underlying reason is unclear [2, 5–8]. The proportion of siblings with type 1 diabetes varies only a little between studies. According to the Finnish Pediatric Diabetes Register study, 1.9% of siblings of newly diagnosed children with type 1 diabetes already had the disease, and, in our previous reports, 2.2% of children who participated in the Finnish Type 1 Diabetes Prediction and Prevention (DIPP) study and progressed to type 1 diabetes had a sibling with type 1 diabetes at the time of data collection [8, 9].

Type 1 diabetes has a subclinical presymptomatic phase identified by the presence of circulating islet autoantibodies [10]. Prospective birth cohort studies have demonstrated that children who are genetically at risk often develop islet autoimmunity very early, at between 9 months and 2 years of age [11–13]. However, the time from islet autoimmunity to stage 3 type 1 diabetes varies widely [10, 14–17]. Progression time depends on the age at seroconversion, longitudinal islet autoantibody profile, sex and genetic background [10, 17–20]. Progression to diabetes is clearly dependent on the duration of multipositivity, as each healthy year with autoantibodies decreased the probability of developing clinical diabetes before puberty by 30% [13].

We hypothesise that, among children with HLA-conferred increased risk for type 1 diabetes, islet autoimmunity during presymptomatic type 1 diabetes differs between those with and without an affected first-degree relative (FDR). It is important to understand the contribution of family history in the disease process of type 1 diabetes when designing clinical screening programmes and intervention trials. Characteristics of the autoimmune process in children with an affected mother or an affected father may be different. The aim of this study was to investigate the initiation and evolution of islet autoimmunity and progression to stage 3 type 1 diabetes in children with at least one FDR with type 1 diabetes (mother, father or sibling) compared to children with no FDR with type 1 diabetes.

## Methods

### Study design

The DIPP study is a population-based prospective follow-up study that was launched in 1994 and recruits all children born in three university hospitals in Finland (Oulu, Tampere and Turku) for screening of class II HLA-conferred genetic susceptibility for type 1 diabetes using cord blood. Children with eligible HLA genotypes associated with increased risk are invited to participate in follow-up until the age of 15 years, with regular measurements of islet autoantibodies from serum samples taken at 3–12 month intervals until the age of 6 years, and at 12–36 month intervals after the age of 6 years. The presence of ICA was used as the primary screening tool for islet autoimmunity in children born before 2003. If the child became positive for ICA or progressed to type 1 diabetes, all samples taken during the follow-up period were also analysed for IAA, GADA and IA-2A. Children born since 2003 have regularly been analysed for ICA, IAA, GADA and IA-2A for all their samples. In addition, if the child was found to be positive for any of these four autoantibodies, ZnT8A were also measured. ICA were measured in all follow-up samples until April 2019. Since then, all follow-up samples have regularly been analysed for IAA, GADA, IA-2A and ZnT8A. If the participant was positive for any of these antibodies at age 15 years, but not diagnosed with type 1 diabetes, follow-up was continued at 3–12 month intervals [21].

Information on family history of type 1 diabetes among FDRs (i.e. mother, father or sibling/half-sibling) was recorded at the time of birth by a structured questionnaire and updated at follow-up visits with an interview by trained nurses (electronic supplementary material [ESM] Methods). Diabetes was diagnosed according to ADA criteria [22].

The study was approved by the Ethics Committee of the Hospital District of Northern Ostrobothnia. All participating families provided written informed consent and the children at an appropriate age gave their assent.

### Study population

**Fig. 1:**
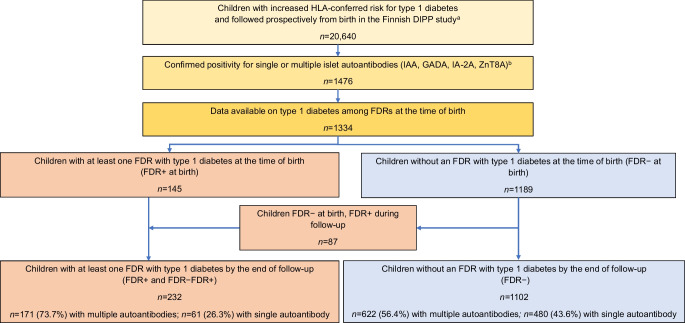
Description of the study population. A total of 20,640 HLA-eligible children have been followed for the appearance of islet autoimmunity in the DIPP study. Positivity for one or more biochemical islet autoantibodies (IAA, GADA, IA-2A and/or ZnT8A) was confirmed in 1476 children, and 1334 of them had data regarding the presence of type 1 diabetes in FDRs. FDR positivity was observed in 145 children at birth, and by the end of the follow-up period, 232 children in total were FDR+ and 1102 remained FDR−. ^a^Data as of 31 August 2022. ^b^Confirmed positivity (i.e. seroconversion) was defined as positivity for the same autoantibodies (single or multiple) in two consecutive samples; data as of 31 December 2022

By the end of December 2022, we had identified 1476 children with confirmed positivity for at least one islet autoantibody (IAA, GADA, IA-2A and/or ZnT8A) out of the total of 20,640 children with HLA-conferred susceptibility who had participated in DIPP follow-up since birth (Fig. 1). The families needed to be able to communicate in either Finnish or Swedish language in order to understand the study information given by recruiting study staff after birth and written in the informed consent form. Data on ethnicity and socioeconomic status were not collected for legal and ethical reasons. Sex was assigned at birth and both boys and girls were equally invited to the study. The newborn population of the catchment area of the three hospitals covers approximately one quarter of all children born in Finland and areas from both southern and northern Finland.

Maternal antibodies were excluded by following the algorithm published previously [21]. Information about type 1 diabetes in the FDRs at the time of birth was available for 1334 (90.4%) of the seroconverted children, and these participants were included in the current analyses.

### Laboratory analyses

Genetic HLA-conferred susceptibility to type 1 diabetes was screened for centrally at the University of Turku using cord blood. Sequence-specific oligonucleotide probes specific for HLA-DQB1*02, DQB1*03:01, DQB1*03:02 and DQB1*06:02/3 alleles were used initially, and genotyping was expanded stepwise to cover more alleles in the DQB1 and DQA1 loci as well as DRB1*04 subtypes [13, 14]. The children in the current study population were categorised into six groups according to the classification of HLA genotypes as conferring high, moderate, slightly increased, neutral or slightly decreased risk for type 1 diabetes, or protection from the disease as described previously (ESM Table 1) [23].

ICA, IAA, GADA and IA-2A were analysed centrally in the Diabetes Research Laboratory at the Department of Pediatrics, University of Oulu. ZnT8A were analysed in the PEDIA laboratory, University of Helsinki. DIPP participants who seroconverted to positivity for any of the autoantibodies (ICA, IAA, IA-2A, GADA or ZnT8A) were scheduled for follow-up visits at 3 month intervals [21]. Seroconversion was defined as the time point at which at least one of the islet autoantibodies was detected for the first time. For the definition of seroconversion for a single autoantibody, we required confirmation for the same autoantibodies in two subsequent samples within 14 days to 14 months. The time of seroconversion for positivity to multiple autoantibodies was defined as the time when at least two autoantibodies (IAA, IA-2A, GADA or ZnT8A) were detected in the same sample for the first time and the same autoantibodies were also observed in the confirmatory sample. For the child to become multipositive after the initial seroconversion, two positive autoantibodies in subsequent samples were required, but the autoantibodies did not have to be of the same specificity.

### Statistical analyses

The difference in the mean follow-up time between the groups was tested using the Mann–Whitney *U* test when there were two comparison groups, and using the Kruskal–Wallis test when the number of groups was more than two. Distributions of categorical variables between the groups were analysed using the exact χ^2^ test. The standard normal distribution (SND) test was used for pairwise comparisons of categorical variables. Logistic regression analysis was used to estimate the risk of various islet autoantibody profiles and progression to type 1 diabetes in children with and without an FDR with the disease. All statistical analyses were performed using IBM SPSS Statistics for Windows, version 29.0 and StatsDirect statistical software, accessed September 2024 (https://www.statsdirect.com/).

## Results

In the study cohort of 1334 seroconverted Finnish children of European origin, the median follow-up time in the DIPP study was 8.6 years (range 0.8–27.0), and the proportion of girls was 39.9% (532/1334). Among the 1334 children 25.2% were positive for multiple autoantibodies at seroconversion and 59.4% were positive for multiple autoantibodies at the end of the follow-up period. A total of 560/1334 (42.0%) developed type 1 diabetes during the follow-up period. At the time of diagnosis, 131 (9.8%) of autoantibody-positive children had an affected FDR.

Of the 1334 seroconverted children, 145 (10.9%) had an FDR with type 1 diabetes at the time of birth, and are referred to as ‘children FDR-positive at birth (FDR+ at birth)’ (Table 1 and Fig. 1). A total of 76 (5.7%) children had a father with type 1 diabetes, 55 (4.1%) had an affected mother, five (0.4%) had two affected parents, and 23 (1.7%) had a sibling with type 1 diabetes (Table 1).

**Table 1:** Family history of type 1 diabetes in 1334 children with confirmed islet autoimmunity in the DIPP study

Family members with type 1 diabetes	FDR+ (*n*=145)	FDR− FDR+ (*n*=87)	FDR+ and FDR− FDR+ combined (*n*=232)
Father only	46.9 (68)^a^	18.4 (16)	29.7 (69)
Mother only	33.8 (49)^b^	2.3 (2)	19.4 (45)
Sibling(s) only	13.1 (19)^c^	75.9 (66)	36.6 (85)
Father and sibling(s)	2.1 (3)^d^	3.4 (3)	8.6 (20)
Mother and sibling(s)	0.7 (1)^e^	0	2.6 (6)
Both parents	3.4 (5)^f^	0	1.7 (4)
Both parents and sibling(s)	0	0	1.3 (3)

Data are shown separately for the 145 children who had an FDR with type 1 diabetes at the time of birth, the 87 children who became FDR+ during the follow-up period, and for all 232 children who were FDR+ at the end of the follow-up period. During the follow-up period, a total of 120 new FDRs were diagnosed with type 1 diabetes, 33 in the families of children who were FDR+ at birth and 87 in the families of children who were FDR− at birth. Of the 120 new diagnoses, 25 were in siblings in the families where other sibling(s) already had type 1 diabetes. Two fathers were diagnosed with latent autoimmune diabetes in adults during the follow-up period, and were included in the type 1 diabetes group. Values are % (*n*)

^a^At the end of the follow-up period, 14/68 transferred into the category of children with a father and sibling with type 1 diabetes, and 1/68 transferred into the category of children for whom both parents have type 1 diabetes

^b^At the end of the follow-up period, 5/49 transferred into the category of children with a mother and sibling with type 1 diabetes, and 1/49 transferred into the category of children for whom both parents have type 1 diabetes

^c^At the end of the follow-up period, all 19 children remained in the same category

^d^At the end of the follow-up period, all three children remained in the same category

^e^At the end of the follow-up period, this child remained in the same category

^f^At the end of the follow-up period, 3/5 transferred into the category of children for whom both parents and sibling have type 1 diabetes

During the DIPP follow-up period, a family member of 87 additional children was diagnosed with type 1 diabetes, and these children are referred to as ‘children FDR-negative at birth, becoming FDR-positive during the follow-up period (FDR− FDR+)’ (Table 1 and Fig. 1). At the end of the follow-up period, 232 (17.4%) of the seroconverted children had at least one FDR with type 1 diabetes, 96 (7.2%) had a father with type 1 diabetes, 58 (4.3%) had an affected mother, seven (0.5%) had two affected parents, and 114 (8.5%) had a sibling with type 1 diabetes. There were 1102 children who did not have an FDR with type 1 diabetes at the end of the follow-up period, and they are referred to as ‘FDR-negative (FDR−)’.

We compared the islet autoantibody profiles at seroconversion and during the follow-up period after seroconversion in the three groups of children: FDR+, FDR− FDR+ and FDR− children. At seroconversion, there was a statistically significant difference between the three groups for GADA positivity and multipositivity (Table 2). In pairwise comparisons, at seroconversion, FDR+ children had a higher risk for GADA positivity and multipositivity than FDR− children, but no difference was observed between FDR− FDR+ and FDR− children (Table 3). During the follow-up period after seroconversion, the three groups differed significantly in the frequency of positivity for IAA, GADA, IA-2A and multiple autoantibodies, but not for ZnT8A (Table 2). In pairwise comparisons, the risk of positivity for GADA, IA-2A and multipositivity was higher in FDR+ children than FDR− children, while FDR− FDR+ children had an increased risk also for IAA positivity compared with the FDR− group (Table 3). The frequency of clinical diagnosis of diabetes was significantly different between the three groups (Table 2), and the risk of diabetes was significantly higher in both FDR+ and FDR− FDR+ children compared with those who were FDR− (Table 3). Neither the age at seroconversion nor the age at diagnosis of type 1 diabetes differed between the three groups (Table 2). However, the median time from seroconversion to diagnosis of type 1 diabetes was significantly shorter in FDR+ children compared with FDR− children (2.7 vs 3.6 years; *p*=0.016) (Table 2). There were no differences in the distribution of HLA-DQB1 genotypes between FDR+, FDR− FDR+ and FDR− children (ESM Table 1).

**Table 2:** Characteristics of 1334 children with confirmed islet autoimmunity in the DIPP study

		FDR+ (*n*=145)	FDR− FDR+ (*n*=87)	FDR− (*n*=1102)	*p* value
At seroconversion	IAA+	57.2 (83)	63.2 (55)	53.1 (585)	0.143
	IAA only	24.8 (36)	29.9 (26)	33.1 (365)	0.120
	GADA+	62.8 (91)	59.8 (52)	52.5 (578)	0.036
	GADA only	33.8 (49)	33.3 (29)	34.3 (378)	0.978
	IA-2A+	17.2 (25)	14.9 (13)	15.6 (172)	0.862
	ZnT8A+	11.7 (17)	10.3 (9)	11.5 (127)	0.940
	Multipositive	35.2 (51)	31.0 (27)	23.3 (257)	0.004
	Age at seroconversion (years)	2.7 (1.5–4.8)	2.1 (1.4–5.7)	3.0 (1.5–5.3)	0.734
During follow-up	IAA+	71.7 (104)	81.6 (71)	68.6 (756)	0.037
	GADA+	85.5 (124)	85.1 (74)	73.5 (810)	0.001
	IA-2A+	67.6 (98)	64.4 (56)	53.3 (587)	0.001
	ZnT8A+	49.0 (71)	54.0 (47)	44.2 (487)	0.141
	Multipositive	73.8 (107)	73.6 (64)	56.4 (622)	<0.001
	T1D diagnosis	55.9 (81)	57.5 (50)	38.9 (429)	<0.001
	Time from seroconversion to next autoantibodies (years)	0.6 (0.3–1.1)	0.5 (0.3–1.4)	0.5 (0.3–1.3)	0.700
	Time from seroconversion to diabetes (years)	2.7 (1.4–5.0)	3.8 (1.5–6.5)	3.6 (1.7–6.4)	0.051^a^
	Age at diagnosis (years)	5.8 (3.7–9.1)	6.4 (3.0–11.2)	6.6 (3.8–10.3)	0.205

Comparison between three groups of children who had an FDR with type 1 diabetes at the time of birth, those who had an FDR who was diagnosed with type 1 diabetes during the follow-up period, and those who did not have an FDR with type 1 diabetes at birth or during the follow-up period. IAA+ may also include other autoantibodies at the same time; IAA only means that IAA is the only autoantibody at seroconversion. The exact χ^2^ test was used for categorical variables, and the Kruskal–Wallis test was used for continuous variables. Data at seroconversion and during the follow-up period are shown separately. Data are % (*n*) for categorical variables and median (IQR) for continuous variables

^a^When comparing two groups, the time from seroconversion to diabetes was significantly different between children who had an affected FDR at the time of birth and those who did not have such a relative either at birth or during the follow-up period (*p*=0.016, Mann–Whitney *U* test)

T1D, type 1 diabetes

**Table 3:** The risk of positivity for various islet autoantibodies at the time of seroconversion and during the follow-up period, and the risk of progression to type 1 diabetes in 1334 children with confirmed islet autoimmunity categorised by the history of type 1 diabetes among their FDRs at the time of birth of the index child or during the follow-up period

		FDR+ vs FDR−	*p* value	FDR− FDR+ vs FDR−	*p* value
At seroconversion	IAA+	1.2 (0.8, 1.7)	0.356	1.3 (0.8, 2.1)	0.181
	GADA+	1.5 (1.1, 2.2)	0.020	1.3 (0.9, 2.1)	0.201
	IA-2A+	1.1 (0.7, 1.7)	0.612	1.0 (0.5, 1.6)	0.937
	ZnT8A+	1.0 (0.5, 1.7)	0.944	0.8 (0.3, 1.5)	0.484
	Multipositive	1.8 (1.2, 2.6)	0.002	1.5 (0.9, 2.4)	0.106
During follow-up	IAA+	1.2 (0.8, 1.7)	0.472	2.0 (1.2, 3.5)	0.014
	GADA+	2.1 (1.3, 3.4)	0.002	2.8 (1.1, 3.7)	0.021
	IA-2A+	1.8 (1.3, 2.7)	0.001	1.6 (1.0, 2.5)	0.049
	ZnT8A+	1.2 (0.8, 1.7)	0.227	1.2 (0.8, 1.8)	0.361
	Multipositive	2.2 (1.5, 3.2)	<0.001	2.1 (1.3, 3.5)	0.002
	T1D diagnosis	2.0 (1.4, 2.8)	<0.001	2.1 (1.4, 3.3)	<0.001

Values are OR (95% CI) by logistic regression analysis

T1D, type 1 diabetes

When comparing FDR+ children who had either a father or a mother with type 1 diabetes at the time of birth, children with an affected father were more often multipositive at seroconversion (39.4% vs 20.0%; *p*=0.033), but the age at seroconversion was similar (Table 4). In addition, the time from seroconversion to the appearance of the next autoantibody specificity was significantly shorter in children with paternal type 1 diabetes (*p*=0.033), and the time from seroconversion to clinical diabetes also tended to be shorter but this difference was not significant (*p*=0.083). However, the age at diagnosis did not differ between the two groups (Table 4).

**Table 4:** Islet autoimmunity and progression to type 1 diabetes in children with either a mother or a father with type 1 diabetes at the time of birth

		Children having a father with T1D at birth (*n*=71)	Children having a mother with T1D at birth (*n*=50)	*p* value
At seroconversion	IAA+	62.0 (44)	50.0 (25)	0.251
	IAA only	25.4 (18)	30.0 (15)	0.568
	GADA+	62.0 (44)	56.0 (28)	0.527
	GADA only	31.0 (22)	42.0 (21)	0.546
	IA-2A+	19.7 (14)	14.0 (7)	0.421
	ZnT8A+	12.7 (9)	10.0 (5)	0.649
	Multipositive	39.4 (28)	20.0 (10)	0.033
	Age at seroconversion (years)	2.1 (1.5–4.6)	2.9 (1.5–4.5)	0.577
During follow-up	IAA+	74.6 (53)	64.0 (32)	0.402
	GADA+	81.7 (58)	92.0 (46)	0.261
	IA-2A+	69.0 (49)	70.0 (35)	0.381
	ZnT8A+	52.1 (37)	42.0 (21)	0.258
	Multipositive	77.5 (55)	68.0 (34)	0.410
	T1D diagnosis	60.6 (43)	50.0 (25)	0.249
	Time from seroconversion to next autoantibodies (years)	0.5 (0.3–0.9)	0.7 (0.5–1.1)	0.033
	Time from seroconversion to diagnosis (years)	2.7 (1.2–4.9)	3.1 (2.1–7.4)	0.083
	Age at diagnosis (years)	6.1 (4.1–8.8)	6.1 (3.7–9.9)	0.476

Three children had a father and a sibling with T1D, and one child had a mother and a sibling with T1D. There were five children whose mothers and fathers both had T1D (not included in this analysis). The exact χ^2^ test was used for categorical variables, and the Mann–Whitney *U* test was used for continuous variables. Data are % (*n*) for categorical variables and median (IQR) for continuous variables

T1D, type 1 diabetes

Positivity for different autoantibodies at seroconversion and during the follow-up, age at seroconversion, time from seroconversion to next autoantibodies and to diagnosis, number of diagnoses, and age at diagnosis were analysed in groups of children with only a father, only a mother or only a sibling with type 1 diabetes at the time of birth, i.e. among children with only one FDR with type 1 diabetes. The children with either a father or a sibling with type 1 diabetes were more often multipositive at seroconversion than those who had a mother with type 1 diabetes. The children with an affected sibling had the highest frequency of multipositivity at seroconversion (ESM Table 2).

During the follow-up period, a total of 793 (59.4%) children developed multiple islet autoantibodies (two or more). When the 793 multipositive children were categorised as either FDR+ or FDR− at birth, there were no differences in the autoantibody status at seroconversion or during the subsequent follow-up period, but FDR+ children had significantly shorter time from seroconversion to clinical diagnosis (3.0 vs 4.1 years; *p*=0.032) (ESM Table 3).

Among the 232 children who were FDR+ by the end of the follow-up period, 171 (73.7%) developed multipositivity, whereas the proportion of multipositive children was lower (622/1102; 56.4%) among children who remained FDR− at the end of the follow-up period (Table 2 and Fig. 1). Among multipositive children, there were no statistically significant differences in the initiation or evolution of islet autoimmunity, or progression to clinical type 1 diabetes between FDR+, FDR− FDR+ and FDR− children. Of note, in contrast to FDR+ children, whose progression from seroconversion to diabetes was the shortest (3.0 years), FDR− FDR+ children had the longest progression time (4.8 years) (ESM Table 4).

## Discussion

We analysed the effect of familial type 1 diabetes on the evolution of islet autoantibody patterns in 1334 children with increased HLA-conferred risk for type 1 diabetes. All participants had seroconverted to positivity for at least one biochemical autoantibody, and 42.0% of them progressed to type 1 diabetes by the end of the follow-up period. If the child had a mother, father or sibling with type 1 diabetes at the time of birth (FDR+ children), the risk of GADA positivity or multipositivity at seroconversion was higher compared to children without such an affected family member (FDR− children). After seroconversion, FDR+ children had higher risk of progressing to multipositivity and clinical diabetes, and their progression time from seroconversion to diabetes was significantly shorter than in FDR− children. If the child was FDR− at birth but a family member was diagnosed with type 1 diabetes later (FDR− FDR+ children), the child’s risk of developing multiple islet autoantibodies and progressing to clinical diabetes was also increased compared with FDR− children, but their progression time was similar to that in FDR− children, which is a novel finding and has implications for monitoring of autoantibody-positive children.

A total of 145 (10.9%) of these HLA-susceptible children who had confirmed islet autoimmunity were FDR+ at the time of birth, but the proportion of FDR+ children increased during the DIPP follow-up period to 17.4% (232/1334 children). This increase during the follow-up period was higher than expected, possibly due to the enrichment of high-risk HLA-DQB1 genotypes in the DIPP study cohort. In the DiMe study, which invited all newly diagnosed children in Finland between 1986 and 1989, the proportion of children with an FDR with type 1 diabetes was 11.2% at the time of diagnosis, and this increased to 15.1% after a median follow-up period of 7.7 years [4]. According to data obtained from the Finnish Pediatric Diabetes Register study, 9.9–10.4% of newly diagnosed children have an FDR with type 1 diabetes at the time of diagnosis [8, 24]. Among our study participants who progressed to type 1 diabetes, the frequency of FDR positivity was very similar (9.8%). However, the FDR status of children with type 1 diabetes has been determined at different time points in different studies. In the TEDDY study, 9.3% of children with a high HLA risk were FDR+ after 7–15 years of follow-up from birth [25].

In accordance with the findings of the Finnish Pediatric Diabetes Register study [8], we observed that paternal type 1 diabetes was more often present before the birth of the child than maternal or sibling disease. At birth, a total of 1.7% of the children in our cohort had a sibling with type 1 diabetes; this proportion increased considerably up to 8.5% by the end of the follow-up period. In the Finnish Pediatric Diabetes Register study,1.9% had an affected sibling at the time of diagnosis, which is in line with our data at the time of birth, but much lower compared with our follow-up data. The difference between these study cohorts is probably caused by the fact that the participants in the Finnish Pediatric Diabetes Register study represent a population not selected by their HLA status.

It is well-known that children who develop more than one islet autoantibody have a higher risk of progression to stage 3 type 1 diabetes than those who remain positive for a single autoantibody [10, 17]. In our series, in which all children had seroconverted, 73.7% of children with a positive family history of type 1 diabetes developed multiple islet autoantibodies, compared with 56.4% of those who had negative family history of the disease, indicating that FDR positivity is associated with the spread of islet autoimmunity. In line with our findings, a family history of type 1 diabetes was shown to be associated with the risk of development of a second autoantibody and multipositivity in the TEDDY study, which included only children with high-risk HLA genotypes [25]. However, additional factors such as infections, dietary and other environmental attributes may be enriched in the affected families, contributing to the disease process of type 1 diabetes [25].

In the present study, FDR+ children had increased frequency of GADA at seroconversion and during the follow-up period compared with FDR− children, but not an increased frequency of IAA, whereas in the BABYDIAB study with non-HLA-selected FDR+ children, IAA was reported to be the most frequently occurring first autoantibody. No comparison was possible with FDR− children as such children were not included in the BABYDIAB study [26]. During the follow-up period, we observed that IA-2A was more common in the FDR+ children than in those who remained FDR−. Positivity for IA-2A is known to be related to high risk for progression to type 1 diabetes [12, 14, 27, 28], and accordingly, we observed that FDR+ children progressed to diabetes faster and more often than FDR− children.

Children with a father who has type 1 diabetes had faster spread of islet autoimmunity and were almost twice as often multipositive at seroconversion compared to those with a mother who has type 1 diabetes. This is in line with earlier observations that paternal type 1 diabetes transfers a higher disease risk to the child than maternal type 1 diabetes [5, 7]. In the BABYDIAB study [29, 30], the children of fathers with type 1 diabetes developed autoantibodies earlier and with higher prevalence than the children of mothers with type 1 diabetes, but we did not observe any difference in age at seroconversion between the two groups in the present study. The results of our study and those of the BABYDIAB study support the concept of relative protection by maternal type 1 diabetes against the appearance of islet autoantibodies compared with paternal type 1 diabetes [29, 30].

In terms of the timing of type 1 diabetes in the FDRs (before or after the birth of the index child), we observed some differences between the three groups (FDR+, FDR− FDR+ and FDR− children). FDR− FDR+ children did not differ from FDR− children at seroconversion, but showed a significantly higher risk of being positive for IAA, GADA, IA-2A or multiple autoantibodies during the follow-up period than FDR− children (Table 3). Interestingly, FDR− FDR+ children had the youngest seroconversion age, but their time from seroconversion to diabetes was similar to that in the FDR− children and appeared longer than in FDR+ children. These data are important to consider in the prediction of the time to diagnosis.

In the future, the FDR background should be considered in screening programmes, because positive family history of type 1 diabetes at birth or during the follow-up period is associated with higher risk of multipositivity and type 1 diabetes. Furthermore, children who are identified as multipositive and were FDR+ since birth have a faster progression from seroconversion to clinical disease compared with multipositive children who were FDR− at birth. Therefore, information about type 1 diabetes among FDRs is important when the child is identified as positive for islet autoantibodies, particularly in stage 1 type 1 diabetes (multipositivity). Furthermore, when asking about family history of type 1 diabetes, it is important to know when the child became FDR+, because those who are FDR− at birth but whose family member develops type 1 diabetes later have a similar progression rate to children who remain FDR−. All in all, FDR+ children should be the focus when planning monitoring and education programmes for autoantibody-positive children and their families.

The strength of this study is the prospective study design and systematic data collection. Through ongoing newborn recruitment and successful, long-term follow-up, the DIPP study has identified a very large number of children and adolescents with confirmed islet autoimmunity, which facilitates investigation of various factors contributing to the heterogeneous pathogenesis of type 1 diabetes. A weakness of this study is the fact that all participants carried increased HLA-conferred risk for type 1 diabetes, which may limit the generalisability of the results. The information on type 1 diabetes in family members was obtained by a structured questionnaire or with an interview by a trained nurse (ESM Methods).

In conclusion, through the largest prospective analysis to date, we demonstrate that in HLA-susceptible children with islet autoimmunity, having an FDR with type 1 diabetes is associated with more frequent positivity for GADA and multiple autoantibodies at seroconversion, more frequent development of IAA, GADA and IA-2A positivity during the follow-up period, and more frequent and faster progression to clinical type 1 diabetes compared to children without an affected FDR. A higher frequency of multipositivity at seroconversion and faster disease progression was observed in children with paternal type 1 diabetes compared to those with maternal type 1 diabetes. These findings suggest that children with a positive family history of type 1 diabetes represent a special group of participants in general population screening programmes and intervention trials.

## Supplementary Information

Below is the link to the electronic supplementary material.

ESM (PDF 772 KB)

## Data Availability

The data that support the findings of this study are not openly available for reasons of sensitivity but are available from the corresponding author upon reasonable request.

## Abbreviations

DIPP: Finnish Type 1 Diabetes Prediction and Prevention
FDR: First-Degree relative
