# Supplementary material for: Islet autoimmunity and progression to type 1 diabetes in the Finnish DIPP study: comparison between genetically susceptible children with and without an affected first-degree relative
Source: Diabetologia. 2025 Nov 8;69(1):93–102. doi: 10.1007/s00125-025-06573-6 (PMC12686032; doi:10.1007/s00125-025-06573-6)
Supplement: Supplementary file 1 — ESM (PDF 772 KB) [file 125_2025_6573_MOESM1_ESM.pdf]

Electronic Supplementary Materials (ESM)

Salla Kuusela, Jaakko J. Koskenniemi, Toni Valtanen, Tytti Pokka, Taina Härkönen, Jorma Ilonen, Johanna Lempainen, Anni Kyrönniemi, Jorma Toppari, Mikael Knip, Päivi Keskinen, Riitta Veijola:

Islet autoimmunity and progression to type 1 diabetes in the Finnish DIPP study: comparison between genetically susceptible children with and without an affected first-degree relative

ESM Methods

Questionnaire for the parents of the newborn baby (Finnish version)................................ Page 2

Questionnaire for the parents of the newborn baby (English version)................................ Page 4

ESM Table 1. .... Page 6

ESM Table 2. .... Page 7

ESM Table 3. .... Page 8

ESM Table 4. .... Page 9

TUTKIMUSRYHMÄN KAPPALE

Hyvät vanhemmat!

Parhaat onnittelut perheenne uuden tulokkaan johdosta! Pyydämme ystävällisesti, että täytätte tämän lomakkeen, jossa kysytään joitakin tietoja perheenjäsenistänne. Vastatkaa avoimeen kysymykseen tai ympyröikää oikea vaihtoehto tai oikeat vaihtoehdot.

VASTASYNTYNEEN TIEDOT

1. Vauvan sukupuoli

Vauva (A)	1	poika	2	tyttö
Vauva (B)	1	poika	2	tyttö
Vauva (C)	1	poika	2	tyttö

2. Perheemme muiden lasten lukumäärä: _________

3. Vauvan täyssisarusten lukumäärä: _________

4. Onko jollakin seuraavista perheenjäsenistä todettu tyypin 1 diabetes?

Vauvan äidillä	1	kyllä	2	ei
Vauvan isällä	1	kyllä	2	ei
Vauvan täyssisaruksella	1	kyllä	2	ei

VAUVAN SISARUSTEN TIEDOT IKÄJÄRJESTYKSESSÄ VANHIMMASTA LUKIEN

TUTKIMUSRYHMÄN KAPPALE

12. Sisarus 1.

a) Sukupuoli 1 poika 2 tyttö **b)** Syntymävuosi ____________

c) Onko sisaruksella sama äiti ja isä kuin vauvalla? 1 kyllä 2 ei

d) Onko hänellä todettu tyypin 1 diabetes? 1 kyllä, vuonna ________ 2 ei

13. Sisarus 2.

a) Sukupuoli 1 poika 2 tyttö **b)** Syntymävuosi ____________

c) Onko sisaruksella sama äiti ja isä kuin vauvalla? 1 kyllä 2 ei

d) Onko hänellä todettu tyypin 1 diabetes? 1 kyllä, vuonna ________ 2 ei

14. Sisarus 3.

a) Sukupuoli 1 poika 2 tyttö **b)** Syntymävuosi ____________

c) Onko sisaruksella sama äiti ja isä kuin vauvalla? 1 kyllä 2 ei

d) Onko hänellä todettu tyypin 1 diabetes? 1 kyllä, vuonna ________ 2 ei

15. Sisarus 4.

a) Sukupuoli 1 poika 2 tyttö **b)** Syntymävuosi ____________

c) Onko sisaruksella sama äiti ja isä kuin vauvalla? 1 kyllä 2 ei

d) Onko hänellä todettu tyypin 1 diabetes? 1 kyllä, vuonna ________ 2 ei

16. Sisarus 5.

a) Sukupuoli 1 poika 2 tyttö **b)** Syntymävuosi ____________

c) Onko sisaruksella sama äiti ja isä kuin vauvalla? 1 kyllä 2 ei

d) Onko hänellä todettu tyypin 1 diabetes? 1 kyllä, vuonna ________ 2 ei

Mikäli perheessänne on enemmän lapsia, pyytäkää lisäpaperi henkilökunnalta.

English translation of the questionnaire.

Copy for the research site

Dear parents!

Congratulations for the newcomer in your family! Please, fill in this form in which we ask for information about your family members. Answer the open question or circle the correct answer/answers.

Information of the newborn

1. Sex of the baby

Baby (A)	1 boy	2 girl
Baby (B)	1 boy	2 girl
Baby (C)	1 boy	2 girl

2. Number of other children in your family: _____________

3. Number of full siblings of the baby: ____________

4. Has any of the following family members been diagnosed with type 1 diabetes?

Mother of the baby	1 yes	2 no
Father of the baby	1 yes	2 no
Full sibling of the baby	1 yes	2 no

Information of the siblings of the baby ordered by age from the oldest to the youngest

12. Sibling 1.
    a) Sex 1 boy 2 girl b) Year of birth: __________

    c) Does the sibling have the same mother and father as the baby? 1 yes 2 no

    d) Has this sibling been diagnosed with type 1 diabetes?

    1 yes, at which year? _________ 2 no

13. Sibling 2.
    a) Sex 1 boy 2 girl b) Year of birth: __________

    c) Does the sibling have the same mother and father as the baby? 1 yes 2 no

    d) Has this sibling been diagnosed with type 1 diabetes?

    1 yes, at which year? _________ 2 no

14. Sibling 3.
    a) Sex 1 boy 2 girl b) Year of birth: __________

    c) Does the sibling have the same mother and father as the baby? 1 yes 2 no

    d) Has this sibling been diagnosed with type 1 diabetes?

    1 yes, at which year? _________ 2 no

15. Sibling 4.
    a) Sex 1 boy 2 girl b) Year of birth: __________

    c) Does the sibling have the same mother and father as the baby? 1 yes 2 no

    d) Has this sibling been diagnosed with type 1 diabetes?

    1 yes, at which year? _________ 2 no

16. Sibling 1.
    b) Sex 1 boy 2 girl b) Year of birth: __________

    c) Does the sibling have the same mother and father as the baby? 1 yes 2 no

    d) Has this sibling been diagnosed with type 1 diabetes?

    1 yes, at which year? _________ 2 no

If there are more children in your family, please, ask an additional form from the personnel.

ESM Table 1. Children's distribution to different HLA risk groups by FDR status at the end of follow-up[a]

**HLA risk groups**[b]	**FDR+ (*n*=145)**	**FDR-FDR+ (*n*=87)**	**FDR- (*n*=1101)**[c]
Strongly decreased risk	0	0	0.3 (3)
Slightly decreased risk	0	1.1 (1)	0.7 (8)
Neutral risk	0.7 (1)	1.1 (1)	2.3 (25)
Slightly increased risk	12.4 (18)	12.6 (11)	17.1 (188)
Moderately increased risk	56.6 (82)	55.2 (48)	54.5 (600)
Highly increased risk	30.3 (44)	29.9 (26)	25.2 (277)

[a]The exact χ^2 test was used to compare the distribution of HLA risk groups of slightly, moderately, and highly increased risk for type 1 diabetes between the three groups of children, *p*=0.370

[b]HLA risk grouping according to Ilonen et al. Genetic susceptibility to type 1 diabetes in childhood-estimation of HLA class II associated disease risk and class II effect in various phases of islet autoimmunity. Pediatr Diabetes 2016;17 (Suppl 22):8-16. doi: 10.1111/pedi.12327. (23)

[c]*n*=1333, one participant had no information about FDR status during the follow-up.

Values are % (*n*)

ESM Table 2. Islet autoimmunity and progression to type 1 diabetes in children with only a father, only a mother, or only a sibling with type 1 diabetes at the time of birth

		Children having only a father with T1D at birth (*n*=68)	Children having only a mother with T1D at birth (*n*=49)	Children having only a sibling with T1D at birth (*n*=19)	*p* value
**At seroconversion**	IAA+	61.8 (42)	51.0 (25)	52.6 (10)	0.492
	GADA+	61.8 (42)	55.1 (27)	78.9 (15)	0.195
	IA-2A+	20.6 (14)	14.3 (7)	21.1 (4)	0.717
	ZnT8A+	13.2 (9)	10.2 (5)	15.8 (3)	0.823
	Multipositive	39.7 (27)	20.4 (10)	52.6 (10)	0.021[a]
	Age at seroconversion (years)	2.1 (1.5-4.7)	2.9 (1.5-4.3)	2.2 (1.4-5.0)	0.870
**During follow-up**	IAA+	73.5 (50)	65.3 (32)	78.9 (15)	0.491
	GADA+	80.9 (55)	91.8 (45)	84.2 (16)	0.245
	IA-2A+	69.1 (47)	71.4 (35)	63.2 (12)	0.816
	ZnT8A+	52.9 (36)	42.9 (21)	63.2 (12)	0.285
	Multipositive	76.5 (52)	69.4 (34)	78.9 (15)	0.659
	T1D diagnosis	61.8 (42)	51.0 (25)	52.6 (10)	0.492
	Time from seroconversion to next autoantibodies (years)	0.5 (0.3-0.9)	0.7 (0.5-1.1)	1.0 (0.3-1.4)	0.099
	Time from seroconversion to diagnosis (years)	2.7 (1.2-5.0)	3.1 (2.1-7.4)	2.4 (0.7-3.8)	0.201
	Age at diagnosis (years)	6.2 (4.1-8.8)	6.1 (3.7-9.9)	4.8 (1.1-7.0)	0.328

The exact χ^2 test was used for comparison of categorical variables and Kruskal-Wallis test was used for comparison of continuous variables.

[a]Pairwise differences, standard normal distribution (SND) test: father only vs. mother only, *p*=0.027; father only vs. sibling only, *p*=0.314; mother only vs. sibling only, *p*=0.009

Data are % (*n*) for categorical variables and median (IQR) for continuous variables

T1D, type 1 diabetes

ESM Table 3. Islet autoimmunity and type 1 diabetes in 793 children with confirmed multipositivity in the DIPP Study.

		**FDR+ at birth and multipositive (*n*=107)**	**FDR- at birth and multipositive (*n*=686)**	***p* value**
**At seroconversion**	IAA+	67.3 (72)	59.9 (411)	0.146
	GADA+	62.6 (67)	59.8 (410)	0.575
	IA-2A+	22.4 (24)	22.9 (157)	0.917
	ZnT8A+	14.0 (15)	15.5 (106)	0.701
	Multipositive	47.7 (51)	41.5 (285)	0.234
	Age at seroconversion (years)	2.0 (1.3-3.9)	2.0 (1.2-3.5)	0.844
**During follow-up**	IAA+	85.0 (91)	83.8 (575)	0.747
	GADA+	89.7 (96)	91.4 (627)	0.569
	IA-2A+	87.9 (94)	87.0 (597)	0.813
	ZnT8A+	64.5 (69)	70.4 (483)	0.215
	Type 1 diabetes	67.3 (72)	63.4 (435)	0.437
	Time from seroconversion to next autoantibodies (years)	0.6 (0.3-1.0)	0.5 (0.3-0.8)	0.442
	Time from seroconversion to diagnosis (years)	3.0 (2.1-5.4)	4.1 (2.2-6.9)	0.032
	Age at diagnosis (years)	6.2 (4.0-9.1)	7.1 (3.9-10.8)	0.153

Comparison between the two groups of multipositive children who had a first-degree relative (FDR) with type 1 diabetes at the time of birth, and those without any FDR with type 1 diabetes at birth is shown. Percentages were compared with standard normal distribution (SND) test and time variables were compared with the Mann-Whitney *U* test. Data are % (*n*) for categorical variables and median (IQR) for continuous variables.

ESM Table 4. Characteristics of 793 children with confirmed multipositivity in the DIPP Study.

		**Children FDR positive at birth** <br>**(*n*=107)**	**Children FDR negative at birth, becoming FDR positive during follow-up** <br>**(*n*=64)**	**Children FDR negative at birth and during follow-up** <br>**(*n*=622)**	***p* value**
**At seroconversion**	IAA+	67.3 (72)	67.2 (43)	59.2 (368)	0.158
	GADA+	62.6 (67)	60.9 (39)	59.5 (370)	0.820
	IA-2A+	22.4 (25)	20.3 (13)	23.2 (144)	0.871
	ZnT8A+	14.0 (15)	14.1 (9)	15.6 (97)	0.881
	Multipositive	47.7 (51)	42.2 (27)	41.3 (257)	0.471
	Age at seroconversion (years)	2.0 (1.3-3.4)	1.5 (1.0-3.4)	2.0 (1.1-3.5)	0.962
**During follow-up**	IAA+	85.0 (91)	90.6 (58)	83.1 (517)	0.828
	GADA+	89.7 (96)	92.2 (59)	91.3 (568)	0.827
	IA-2A+	87.9 (94)	85.9 (55)	87.3 (543)	0.935
	ZnT8A+	64.5 (69)	73.4 (47)	70.1 (436)	0.398
	Type 1 diabetes	67.3 (72)	70.3 (45)	62.7 (390)	0.357
	Time from seroconversion to next autoantibodies (years)	0.6 (0.3-1.0)	0.4 (0.2-1.0)	0.5 (0.3-0.8)	0.721
	Time from seroconversion to diabetes (years)	3.0 (2.1-5.3)	4.8 (1.6-7.2)	4.0 (2.0-6.9)	0.096
	Age at diagnosis (years)	6.2 (3.9-9.0)	7.7 (3.0-11.5)	7.0 (3.8-10.7)	0.356

The three groups of multipositive children who had a first-degree relative (FDR) with type 1 diabetes at the time of birth, those with an FDR diagnosed with type 1 diabetes during the follow-up period, and those without any FDR with type 1 diabetes at birth or during the follow-up period were compared. The exact χ^2 test was used for comparison of categorical variables and the Kruskal-Wallis test was used for comparison of continuous variables. Data are % (*n*) for categorical variables and median (IQR) for continuous variables.
